# An End-of-Life Plastic and Additive Flow Tracker Tool for Scenario Forecasting

**DOI:** 10.1021/acs.iecr.5c00426

**Published:** 2025-06-17

**Authors:** John D. Chea, Matthew Conway, Gerardo J. Ruiz-Mercado, Kirti M. Yenkie

**Affiliations:** # Office of Research & Development, US Environmental Protection Agency, Cincinnati, Ohio 45268, United States; ‡ Department of Chemical Engineering, Henry M. Rowan College of Engineering, 3536 Rowan University Glassboro, New Jersey, 08028, United States; § Chemical Engineering Graduate Program, University of Atlántico, Puerto Colombia 080007, Colombia

## Abstract

Plastics are widely used for their affordability and versatility in various applications. However, the end-of-life (EoL) management stage can often lead to the release of hazardous chemical additives and degradation products into the environment, which leads to ecological and human exposure risks. The increasing demand for plastics is expected to escalate the frequency of material releases during plastic EoL management activities, creating a challenge for policymakers, consumers, manufacturers, and communities to ensure proper material segregation, reuse, recycling, and disposal. Effective management is crucial for achieving a safer and sustainable circular economy (CE) for plastics, enhancing recycling efficiency and promoting material reuse. End-of-life plastic research efforts often overlook chemical additives, which are crucial for understanding the environmental and health implications of plastic usage. Therefore, chemical additive content and release must be assessed and considered when designing and implementing technologies, supply chains, incentives, and regulations for plastic CE management solutions. This research offers a Python-based EoL plastic and additive flow tracker tool (EoLPAFT) to support decision-makers in analyzing the holistic impacts and benefits of potential plastic EoL management solutions, considering the chemical additives within EoL plastics, their releases, and occupational exposure scenarios. The utility of the tool was tested through two hypothetical case scenarios, including (1) nationwide adoption of an extended producer responsibility (EPR) program and (2) maintaining a CE for plastic. The analyses projected by the tool can ease the prediction of long-term outcomes, offering technical knowledge and insight for policymakers and stakeholders seeking to minimize the environmental, social, economic, and health impacts of plastic pollution while seeking a safer and more sustainable CE of plastics considering chemical additives.

## Graphical abstract

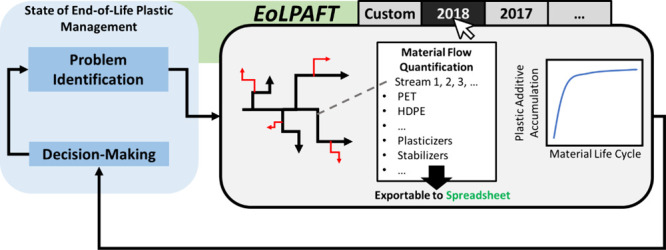

## 1 Introduction

Plastic products are commonly used in many modern-day applications due to their versatility and adaptability.^1^ However, the conventional end-of-life (EoL) plastic management processes can release chemical additives, micro- and nanoplastics, and degradation byproducts into the environment, creating social, health, environmental, and economic concerns.^2–4^ Generally, chemical additives found in plastic products are not chemically linked to the polymer chain and can migrate to the environment based on the molecular weight, temperature, compatibility, and solubility in the surrounding medium.^5–7^ Plastic material tracking efforts focusing on global-level management without considering chemical additive content have previously been reported, showing that business-as-usual approaches could only reduce plastic pollution in the ocean by 7%.^8^ In 2020, it was predicted that adopting more drastic management policies and investing in the necessary technologies could achieve up to 80% reduction in EoL plastics flows into the ocean by 2040.^8^ However, the primary research does not quantify chemical additive flows within the mass allocation, which underemphasizes the impact of plastic releases on the environmental compartments (land, water, and air), the techno-economy feasibility of CE efforts, and the safety of recycled plastic products.^9^ Other research groups have considered the presence of chemical additives in a specific polymer type, but uncertainties remain regarding the identity and quantity of these chemicals.^10^ Chea et al. (2023) analyzed the EoL plastics management processes in the US by completing a material flow analysis, identifying plastics and chemical additives releases and exposure scenarios on safety, human health, and the environment.^11^ However, the completed material flow analysis provided only a snapshot of EoL plastic management in the US in 2018. Investigating other scenarios, such as other years or smaller scales (municipal- or state- rather than national-level) is difficult to estimate with that framework alone. Altering parameters from the reported methodology to explore other scenarios is prone to human errors. Alternatively, manual calculations can be tedious and lead to an even higher possibility of calculation errors. Therefore, incorporating automation with customizable parameters can prove useful to decision makers for testing different scenarios of EoL plastic management and tracking individual itemized components (i.e., mass of different plastic types and chemical additives) to achieve resource sustainability and develop safer and more sustainable plastic CE paths.

This research aims to enhance the material tracking effort by creating a Python-based EoL plastic and additive flow tracker tool (EoLPAFT) to quickly analyze the effects of different factors (such as recycling, incineration, and landfilling rates; plastic composition; and plastic exports and imports) throughout the plastic life cycle and estimate releases, simplifying material flow analysis to support decision-making. This tool offers a holistic overview of plastics and chemical additives flow paths and releases to the environment associated with common EoL plastic management scenarios (i.e., recycling, incineration, and landfilling). Stakeholders, policymakers, and researchers could use this tool to quickly estimate the efficiency of the existing EoL plastic management techniques and test the feasibility of new EoL plastic management strategies and technologies to improve material circularity in the overall plastic management supply chain.

Including the introduction, this article is organized into four sections to describe the capabilities of this automated tool. Section 2 describes the methodology behind the construction of the tool, data needs, and context behind the case study scenarios in the existing EoL plastic management process. These scenarios include (1) theoretical implementation of the Extended Producer Responsibility (EPR) program within the US and (2) tracking plastics across multiple life cycles. Section 3 discusses the major results derived from the tool for the described case studies, such as data, visualization, and scenario forecasting, and section 4 provides final thoughts and conclusions on the usefulness and applications of this tool. Studying the effects of parameter changes to EoL management practices can ensure justifiable alterations to the existing designs of EoL management infrastructure and allow for more informed decision-making toward sustainable EoL plastic management.

## 2 Methods

The Python-based EoLPAFT consists of several integrated components designed to provide an automated material flow analysis for plastics and additives for any given year and generate usable data to assist users in assessing impacts and risks from EoL plastic management. The EoLPAFT is divided into three primary parts: the front end, the back end, and the core functionalities. Figure 1 illustrates a schematic diagram of the essential components of the EoLPAFT. The front end of the tool features a graphical user interface (GUI) that allows users to input basic or preloaded US-specific EoL plastic management data (such as recycling, incineration, and landfilling rates, waste masses, and plastics compositions) without requiring advanced programming knowledge. The back-end handles data processing and material flow calculations using Python libraries such as NumPy and Pandas. This separation of major tool components ensures the tool is modular. The core functionalities of the tool encompass various aspects of EoL plastic management. Key features include (1) tracking the flow of plastics and chemical additives through different EoL scenarios, (2) estimating the release of plastics and additives, (3) comparing the effects of altering plastic management strategies to achieve a desired plastic recovery target, and (4) providing visualizations and reports to support decision-making and further research and development.

**Figure 1:**
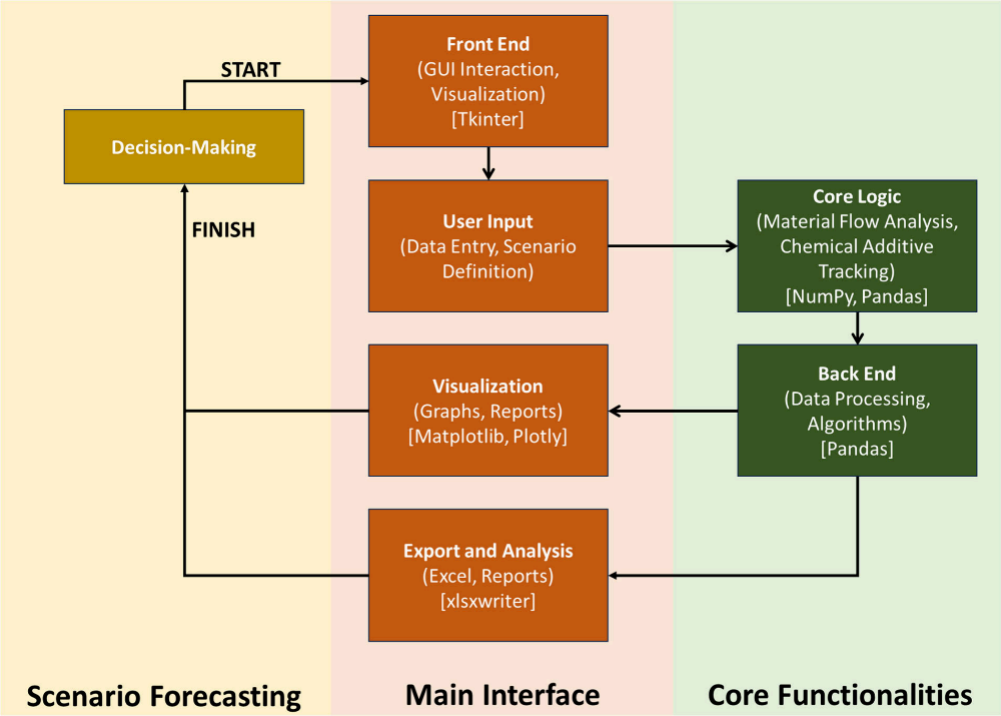
End-of-life plastic and additive flow tracking tool schematics.

### 2.1 Tool Design and Use

The constructed EoLPAFT is intended to provide a holistic insight into the impacts of potential policy implementations using a material flow analysis to estimate the relative mass flow intensity and material releases into the environmental compartments at various stages throughout EoL plastic management. The Python-based graphical user interface (GUI) component was constructed using the Tkinter module, which uses a series of Tkinter entry widgets to take user input for variable data sets. An example of the data entry GUI is shown in Figure 2. The user may create a hypothetical scenario by specifying the total municipal solid waste (MSW) mass and composition (e.g., percent plastic, metal, and food scraps). The plastic mass composition (i.e., percent PET, HDPE, etc.) within the overall EoL plastic mass in MSW must also be specified. The tool will then calculate the masses of the different types of MSW and plastics in the waste stream (i.e., 35.7 million tons within the US in 2018 based on 292 million tons of generated MSW). The allocation of the total EoL plastics generated can be further adjusted between conventional management techniques such as recycling, incineration, and landfilling. Within the recycling effort, the allocation can be further made to differentiate between EoL plastics exported for recycling in other countries and the amount domestically recycled. For materials subjected to domestic recycling, the efficiency of material recovery can be customized. Several interactive buttons are available to assist users with filling in the required input data. The “Check Proportions” button ensures the input data do not exceed 100%. An “Autofill 2018 Data” button automatically populates the data entry with the state of EoL plastic management in 2018, which is intended to be a default value if the user is unsure. Data are submitted using the “Enter Above Dataset” button, and once data entry is complete, the “Calculate Streams” button performs the material flow analysis, itemizing EoL plastics and chemical additives by resin types and function. As a note: because chemical recycling is not widely implemented in the US, it has not been included in this work, which serves as a high-level analysis of common waste management practices.

**Figure 2:**
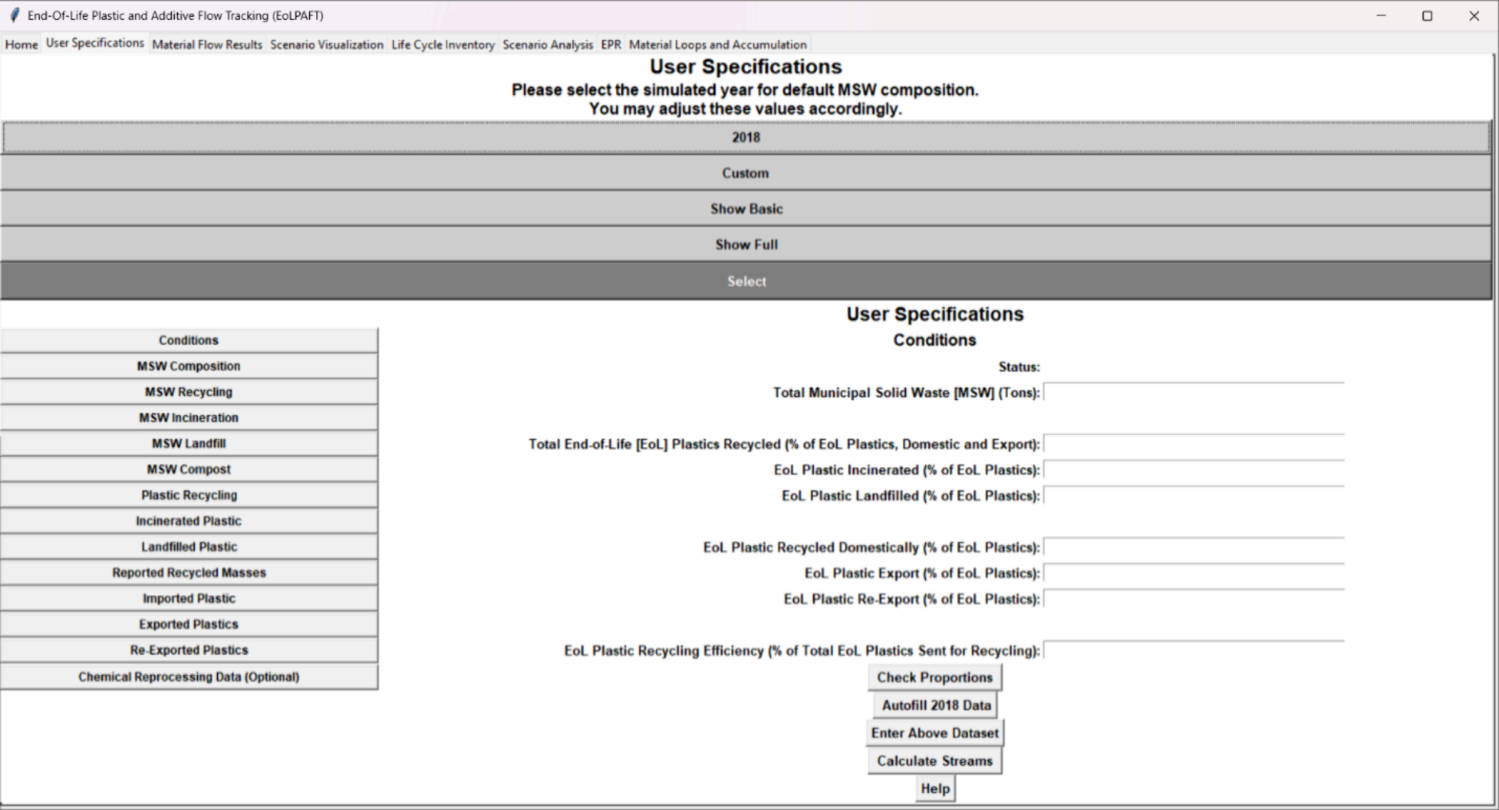
Data entry example using the graphical user interface.

Data handling and analysis within this EoLPAFT utilize NumPy and Pandas. Most information is stored in dictionaries, which pair a key (usually a type of plastic resin or additive) with a value (i.e., mass flow, mass fraction, or other numerical datum). Tkinter widgets display numerical data automatically. Additionally, Matplotlib was used to generate bar and pie charts and scatter plots to aid in the visualization of the different scenario analyses (i.e., EPR and chemical accumulation). Plotly was used to generate a Sankey diagram for material flow illustration, which is automatically saved locally as a .html and .png file. Calculated values from the material flow analysis may be transferred to Microsoft Excel through the xlsxwriter module with a timestamp. This method allows for easy data collection that can be conveniently processed by users unfamiliar with coding without accidentally overwriting an older file. Table 1 summarizes the Python libraries and the rationale for their usage.

**Table 1:** Python Libraries Used to Construct Important Components of the EoLPAFT

Python Libraries	Description/Purpose
Matplotlib	Creating plots/charts for visualization
NumPy	Performing mathematical operations on arrays
Pandas	Data processing and analysis
Plotly	Data visualization (Sankey Diagram) for material tracking
Tkinter	Creating a graphical user interface for data input and result display
Xlxswriter	Writing result files into Microsoft Excel

The user interface is designed to be simple, allowing users to input data relevant to any municipal solid waste (MSW) program. Users can generate artificial input scenarios or use the base case data from 2018 to fill in any missing data sets. The tool explains each data entry field, instructing users on its meaning so they can understand the implications of the input. These parameters (i.e., total EoL plastics generated and plastic recycling rate) may be modified to represent the local communities. To reduce complexity, this tool offers a “Basic View” option that allows users to input sample data from 2018, requiring minimal configurations. Therefore, users with less technical knowledge can grasp basic trends, such as the impacts of increasing plastic recycling rates on total environmental releases. The tool then generates illustrative graphics for the plastics and additive flows and optionally shows the numerical values for each flow path. Therefore, EoLPAFT provides an overview of plastic flows throughout the MSW management system while remaining usable for all stakeholders, regardless of their technical background.

### 2.2 Data Collection and General Methodology

The material flow analysis is based on a bottom-up approach, consolidating and processing the data collected from individual studies to model the complexity of plastic EoL management within the US.^11^ Figure 3 describes the logical methodology for tracking plastics and chemical additives during EoL material management and assessing the environmental implications.

**Figure 3:**
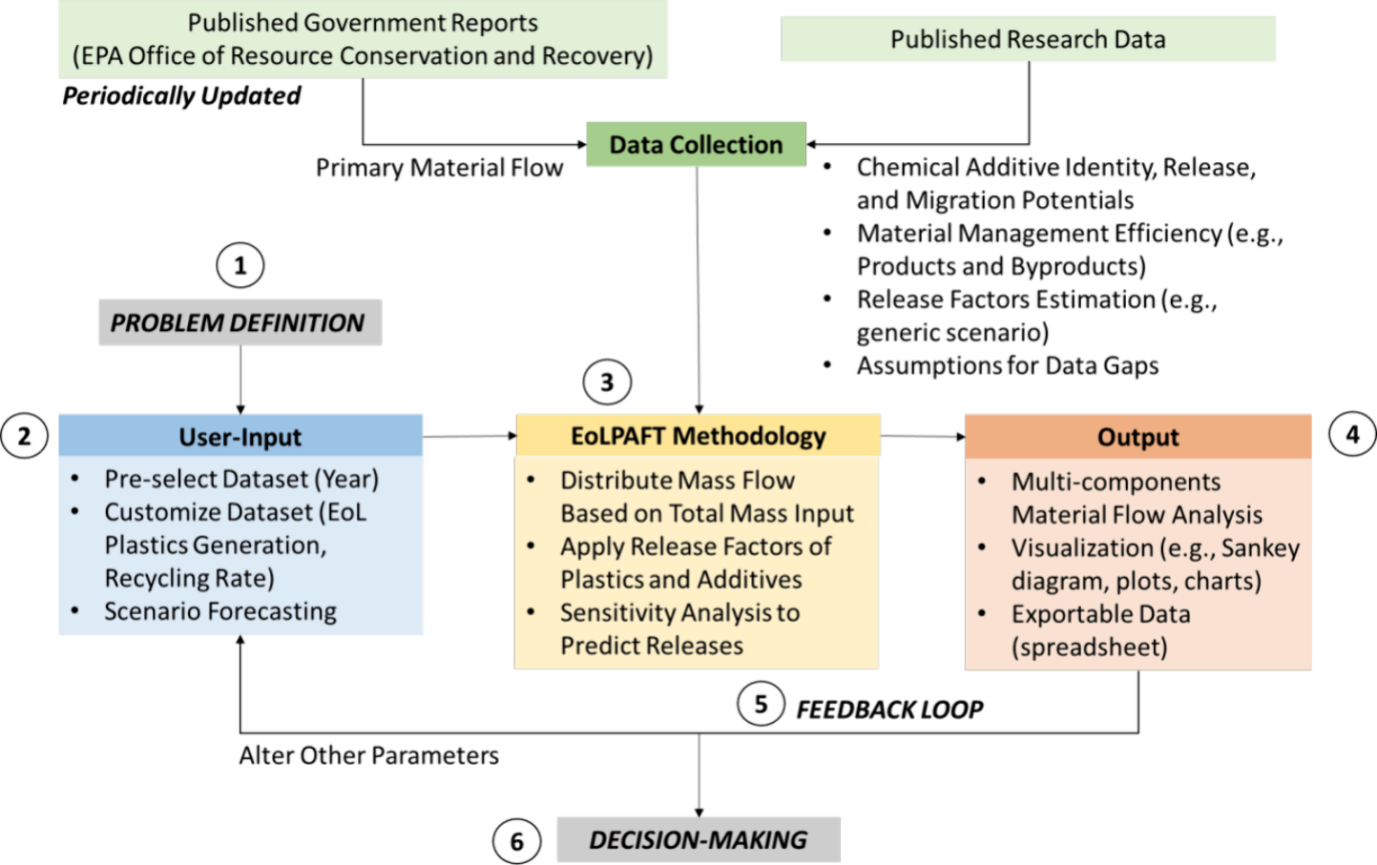
Overview of EoLPAFT and features of the constructed tool in tracking plastics and chemical additives during the end-of-life material management stages for decision-making.

By default, to distribute mass flow the EoLPAFT uses values from publicly available government reports and published research data.^5,12–16^ Notably, the US Environmental Protection Agency (EPA) reported 35.7 million tons of EoL plastic collected in 2018, which included 14.8% polyethylene terephthalate (PET), 17.6% high-density polyethylene (HDPE), 2.4% poly­(vinyl chloride) (PVC), 24.1% low-density polyethylene (LDPE), 0.3% poly­(lactic acid) (PLA), 22.8% polypropylene (PP), 6.3% polystyrene (PS), and 11.7% categorized as other plastics by mass.^15^ The chemical additive content of these plastics may vary based on the intended applications. Table 2 provides the common chemical additives found in various plastics, their chemical classes, and the associated range of usage concentration. The extensive ranges demonstrate the inherent uncertainties caused by the knowledge gap between manufacturing and EoL management. To serve as the basis for the material flow analyses, these data were converted into release factors in terms of the mass of the substance released per unit mass of the input. Users may have a predefined question regarding the EoL plastic management within the US or wish to conduct sensitivity analyses. In the base case analysis, the overall EoL plastic flow allocations are consistent with the 2018 plastic management statistics reported by the US EPA, the most up-to-date data available during this research.^14^ However, users may wish to include unreported events, such as material releases to the environment and chemical additive contamination possibilities. In those instances, users can employ the generic scenario analysis approach by Chea et al. (2023).^11^ The generic scenario analysis identified the major environmental chemical release potentials during the typical EoL pathways and operating conditions, providing release factors for the EoLPAFT methodology. After the analysis is run, the completed material balance is automatically tabulated into an exportable spreadsheet, followed by a visual representation of the relative material flow allocation. The Python code that executes the described methodology can be found in a GitHub repository at https://github.com/jdchea95/EoLPAFT.

**Table 2:** Common Chemical Additives Found in Plastics^a^

Type	Potential Application^a^	Chemical Classes	Composition Range (% wt. of total product)
Plasticizers	PVC, cellulose plastic	Phthalates, short and medium-chain chlorinated paraffins	10–70
Flame Retardants	Foam	Brominated and Chlorinated Flame Retardants	2–28
Antioxidants	LDPE, HDPE, HIPS, ABS	Phenolic Antioxidants, Phosphites	0.05–3
UV Stabilizers	PE, PP, PVC	Henolic Benzotriazoles, Cadmium Compounds, Lead and Lead Compounds	0.05–10
Heat Stabilizers	PVC	Cadmium Compounds, Lead Compounds, Nonylphenol, Barium and Calcium Salts	0.05–3
Slip Agents	LDPE, PP	Erucamide, Oleamide, Stearamide	0.1–3
Lubricants	PVC, PS/ABS, PP, PE	Fatty Acid Esters, Hydrocarbon Waxes, Metal Stearates, Amide Waxes, Ester Waxes	0.1–3
Antistatic Agents	PE films, PE and PP Foams, PVC, PP injection molding application	Long-Chain Alkyl Phenols, Ethyoxylated Amines, Glycerol Esters	0.1–1
Curing Agents	Epoxy resins	4,4′-Diaminodiphenylmethane	0.1–2
Blowing Agents	PVC, PE, epoxy resins	C,C′-azodi(formamide), hydrofluorocarbons, sulfur hexafluoride	0.05–20
Biocides	PUR, PVC	Organic Tin Compounds, Arsenic Compounds, Triclosan	0.001–1
Catalyst	PVC, PE, PP, and other nonspecified plastics, PUR	Chromium and Chromium Compounds, Mercury and Mercury Compounds	0.1–0.3
Colorants	PVC, PE, PP, and other nonspecified plastics	Cadmium Compounds, Chromium Compounds, Lead Chromates	0.01–5
Fillers	Any plastic	Calcium Carbonate, Barium Sulfate, Clay, Talc, Kaolin, Mica	0–50
Reinforcements	Any plastic	Glass Fibers, Carbon Fibers	15–30

a Adapted from Hahladakis et al., Hansen, and the UN Environment Programme.^5,17,18^

b Note: ABS = acrylonitrile butadiene styrene, HDPE = high-density polyethylene, HIPS = high-impact polystyrene, LDPE = low-density polyethylene, PE = polyethylene (high- and low-density), PP = polypropylene, PS = polystyrene, PVC = poly­(vinyl chloride), PUR = polyurethane

### 2.3 Case Scenario Analysis Formulation

The utility of the constructed EoLPAFT was demonstrated through the sensitivity analysis feature, which allows parameter variation to test the effects on responses such as material releases to the environmental compartment. The assessment methodology described in section 2.2 provides a set of base-case results for comparison with alternative scenarios to evaluate the EoL plastic management infrastructure. Two scenarios, in sections 2.3.1–2.3.2, describe the justification and methodology for analyzing the effects of implementing a new EoL plastic management policy nationwide and the continuous recycling of EoL plastics on safety and contamination level concerns due to chemical additive accumulation. These two scenarios were selected because they demonstrate different situations that already exist to some degree or in some jurisdictions and show the ways that commonly discussed policies may affect plastic additive releases. This action corresponds to steps 1 and 2 of Figure 3, demonstrating the thought process required from the users before running the tool.

#### 2.3.1 Extended Producer Responsibility

The European Union (EU) has widely adopted the Extended Producer Responsibility (EPR) program to enhance plastic EoL management strategies and place more accountability on manufacturers for their product EoL stage management. Within the EU EPR program, producers are expected to design products with the eventual intention of recovering in the EoL management stage and finance the recovery efforts through fees. Plastic packaging, in particular, constitutes up to 40.7% of the total plastic EoL material generated in the US in 2018, 13.6% of which was successfully recycled.^15^ The variation in packaging types, collection methods, and existing recycling infrastructure throughout the US contribute to its low recycling rates. Decision-makers may use EoLPAFT to test the effects of implementing an EPR program and identify potential undesired effects (e.g., chemical additive accumulation in recycled plastic products) of meeting specific recycling goals before proposing EPR programs.

As of April 2023, six US states (CA, CO, ME, NJ, OR, and WA) implemented packaging EPR laws that require producers to make packaging recyclable or compostable, achieve higher recycling rates for single-use plastics, reduce plastic packaging volumes, and incorporate postconsumer recycled content into various products.^19^

Although the focus varies between states, these laws are meant to improve the plastic EoL and enable better EoL management practices, with the potential to reduce plastic landfilling, incineration, and hazardous environmental pollution. The existing EPR infrastructure in these states includes (1) having a producer responsibility organization (PRO) collect EoL material, process, and transport EoL material as needed and (2) allowing municipalities to manage EoL material and then be reimbursed by the PRO.^20^ These efforts aim to promote a circular economy (CE), reduce landfill EoL material allocation, encourage sustainable packaging practices, and upgrade recycling systems within each state.

A scenario analysis section was created in the tool to test the effects of an EPR program on the enhancement of plastic packaging recycling and estimate the potential to reduce releases to the environmental compartments. Releases include EoL plastics, chemical additives, and other byproducts, such as micro- and nanoplastics. The methodology assumes that under EPR, manufacturers (typically through PRO) fund the collection, sorting, and mechanical recycling of recoverable EoL plastic packaging, effectively reducing the volume of material processed through other EoL management methods.^21^ This scenario separates 40.7% of packaging plastic into its own stream and varies the recycling rate of this stream. The remaining plastics are handled using conventional EoL management methods without manufacturer involvement. The increased recycling rate is achievable through low recycling costs, which can be aided by cooperation between producers and recyclers.^22^ It should also be noted that the calculated values shown in section 3 are based on plastic EoL management performance from 2018 and do not necessarily represent EoL plastic management from other years.^14^ However, the general trends from this illustrative case are expected to remain consistent unless there is a drastic change in the EoL material management infrastructure and composition of plastic products.

#### 2.3.2 The Impacts of Maintaining Circular Economy

In addition to testing new policies (e.g., EPR), the effects of maintaining continuous plastic physical recycling were estimated across multiple life cycle loops (manufacturing, use, EoL, recycling, and manufacturing). CE is envisioned as a system emphasizing resource conservation and material reuse to promote sustainability principles.^1^ It has been recognized that plastic pollution has been linked as a direct source of micro- and nanoplastics and chemical additives released into the environment.^23^ In theory, achieving CE for the plastic supply chain can greatly reduce plastic pollution; however, a closed-loop CE is not necessarily feasible for plastics because the conventional plastic recycling method (mechanical recycling) can degrade plastics and propagate chemical contamination throughout multiple life cycle loops.^24^ Chemical additives are often introduced as processing aids in physical reprocessing.^11^ Thus, in a circular economy, the recycled plastics may later be compounded with fresh materials and chemical additives in the subsequent product manufacturing life cycle, perpetuating and increasing the contamination of the environment and human health because chemical additives (with now higher concentration values) can increase their transport rates into the surrounding medium (i.e., food and water).^2,5,6,25^ Therefore, understanding the fate of chemical additives throughout the life cycle of plastics can aid in developing strategies to mitigate the adverse effects of in-excess chemical additive quantities present during the plastic manufacturing, use, and EoL pathways.

This assessment was completed through a series of automated life cycle calculations. All parameters are set to represent the EoL plastic management in 2018. The total plastics and chemical additive content recovered through the conventional recycling method are recorded in the initial life cycle (0), then sent to manufacturing in the subsequent life cycle (1) to be compounded with virgin polymers and other additives to create new plastic products. The same iteration is made through multiple life cycles (1, 2, 3, and 4) to examine the dispersal of the original materials into the environmental compartment and introduce chemicals of concern into the plastic supply chain. This scenario assessment strictly tracks the fate of the plastics originating from life cycle 0 to the potential reuse in the new life cycles, and, like the rest of the tool, is a material flow analysis. This study does not model the polymer nor its additives, nor the quality of the recyclate, instead focusing on the mass flow through the life cycles. This assumption maintains a level of simplicity, which holds that, in the best-case scenario, recycled plastics are reprocessed as intended for further use. The associated chemical contamination from maintaining CE of plastics was estimated, and this process is shown graphically in Figure 4.

**Figure 4:**
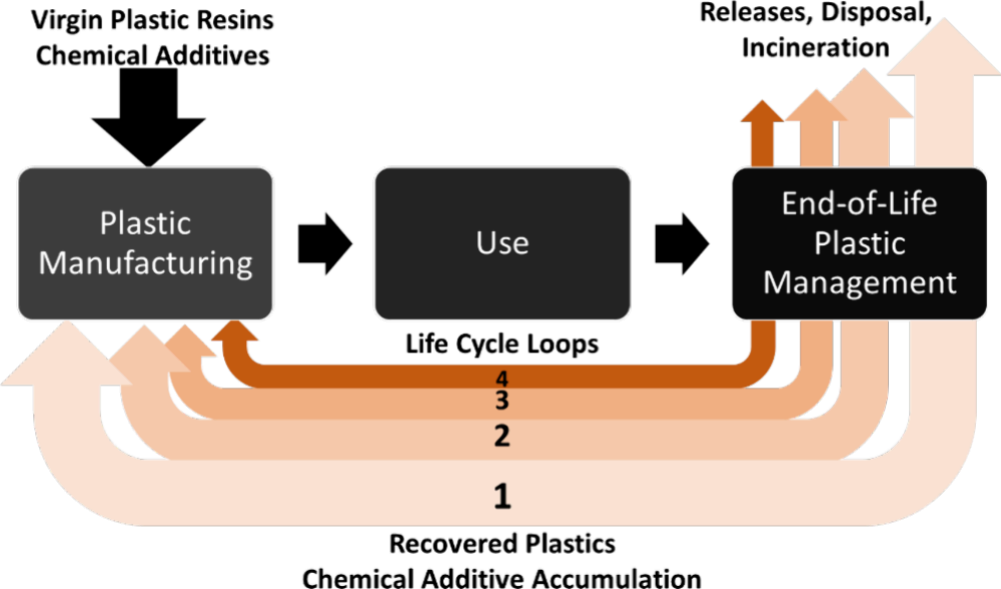
An illustration of chemical additive accumulation potentials caused by continuous recycling of end-of-life plastics.

#### 2.3.3 Uncertainty Analysis

There is notable uncertainty in the mass fractions of additives in plastic products. Each type of additive has a range of common mass percentages (shown in Table 2). Because plastic formulations are proprietary, exact masses and mass percentages cannot be known. Therefore, the effects of this uncertainty were investigated using a Monte Carlo sampling approach for uncertainty simulation to determine the relative impact of the mass percent of each category of additive.

The average mass fraction of each additive category (from Table 2) was assumed to be the average of the high and low values found in the literature (i.e., for plasticizers, the high value was 70%, and the low value was 10%, with an average of 40%). The range given was assumed to be six standard deviations (three above and three below the mean). Then, probabilities were found using a random number generator and then converted to mass fractions by assuming a normal distribution of mass fractions with the given average and standard deviation. These probabilities were then substituted into the EoLPAFT algorithm to replace one additive’s mass fraction at a time (e.g., when a new value for plasticizer was substituted, the low value for all the other additive categories was kept). There were a total of 850 data points accounting for 50 probabilities for all 17 additive categories.

## 3 Results and Discussions

The Python-based EoLPAFT offers a holistic approach to assist government, manufacturer, and academic stakeholders with assessing the impacts of various alterations to end-of-life (EoL) plastic management practices. Users can analyze the potential effects of policy applications, technology development, and changes in management practices throughout the plastic life cycle, simplifying complex assessments into easily interpretable insights. The capability of the tool was demonstrated by evaluating the potential adoption of EPR programs and tracking plastic and additive fates across multiple manufacturing and recycling life cycle loops. Therefore, the benefits and challenges related to the resource conservation of the existing EoL plastic management supply chains and potential sustainable solutions can be quickly revealed, supplying valuable insights for government, manufacturer, and academic stakeholders for designing and evaluating potential more sustainable circular EoL plastic management solutions, incentives, and regulations.

### 3.1 Extended Producer Responsibility

This analysis enables a comparison between existing EoL plastic management practices and the potential effects of EPR programs, specifically focusing on plastic packaging EoL. There is a positive correlation between the recycling rate of packaging plastics and the effective adoption of EPR programs nationwide. Globally, the effectiveness of EPR programs has achieved a packaging recycling rate as high as 75%, while the statistics within the US may be much lower due to the lack of sufficient funding in communities throughout the country.^26^ EPR programs can effectively reduce the number of plastics sent to traditional EoL management by requiring manufacturers to take responsibility for their products throughout the material life cycle. Increased plastic collection and recovery rates and decreased plastic mass in landfills or incineration can also be observed. Therefore, the potential content of plastic packaging chemical additives and their releases along the use and EoL life cycle stages support the need to improve the manufacturing stage of plastic packaging products and minimize potential health and environmental risks.

The case study result for the EPR scenario is shown in Figure 5, which displays the EoLPAFT output regarding the potential material releases to land and water and its decreasing trend with increasing plastic recovery rate. Air releases correlating directly to EoL plastic management are generally negligible compared to land and water releases. Thus, this release has been excluded from further analysis. The recycling rates (%) include practices such as plastic exports and conventional management of nonpackaging plastics, which are unaffected by the EPR programs. Figure 5 shows that in the EPR scenario additive releases to land and water decrease linearly as the recycling rate increases, in contrast with the base case (non-EPR) in which the releases increase. This difference reaches reductions of up to 40% of additive releases at maximum packaging plastic recycling rates in the EPR scenario compared to the non-EPR scenarios. The EPR program can reduce the material released to the environmental compartments with an increasing recycling rate because more EoL plastics are systematically collected and directed into recycling, supported by manufacturer funding. Thus, coordination among stakeholders may be improved relative to the conventional EoL plastic recycling methodology, which can create unintentional releases and contamination, leading to hazardous workplace exposure and unsafe CE paths.^11^ For example, the conventional EoL plastic management processes often involve multiple generic steps (i.e., reprocessing, incineration, landfilling, and transportation), typically without considering product-specific characteristics. Limited information exchange between the manufacturers and waste handlers leads to a lack of targeted management methods and efficiency issues. Consequently, without producer-specific guidance, material processing in the EoL stage typically follows standard municipal or facility-level waste management protocol, including sorting by polymer type, recycling, and using incineration and landfilling for contaminated or nonrecyclable streams. This protocol does not account for product-specific characteristics or chemical composition, potentially subjecting the EoL plastics to excessive processing steps (e.g., processing EoL plastics without end-market, redundant transfer between sorting facilities, incineration, or landfilling of potentially recyclable plastics), leading to increased resource and energy waste, material loss, operational costs, and releases into the environment.

**Figure 5:**
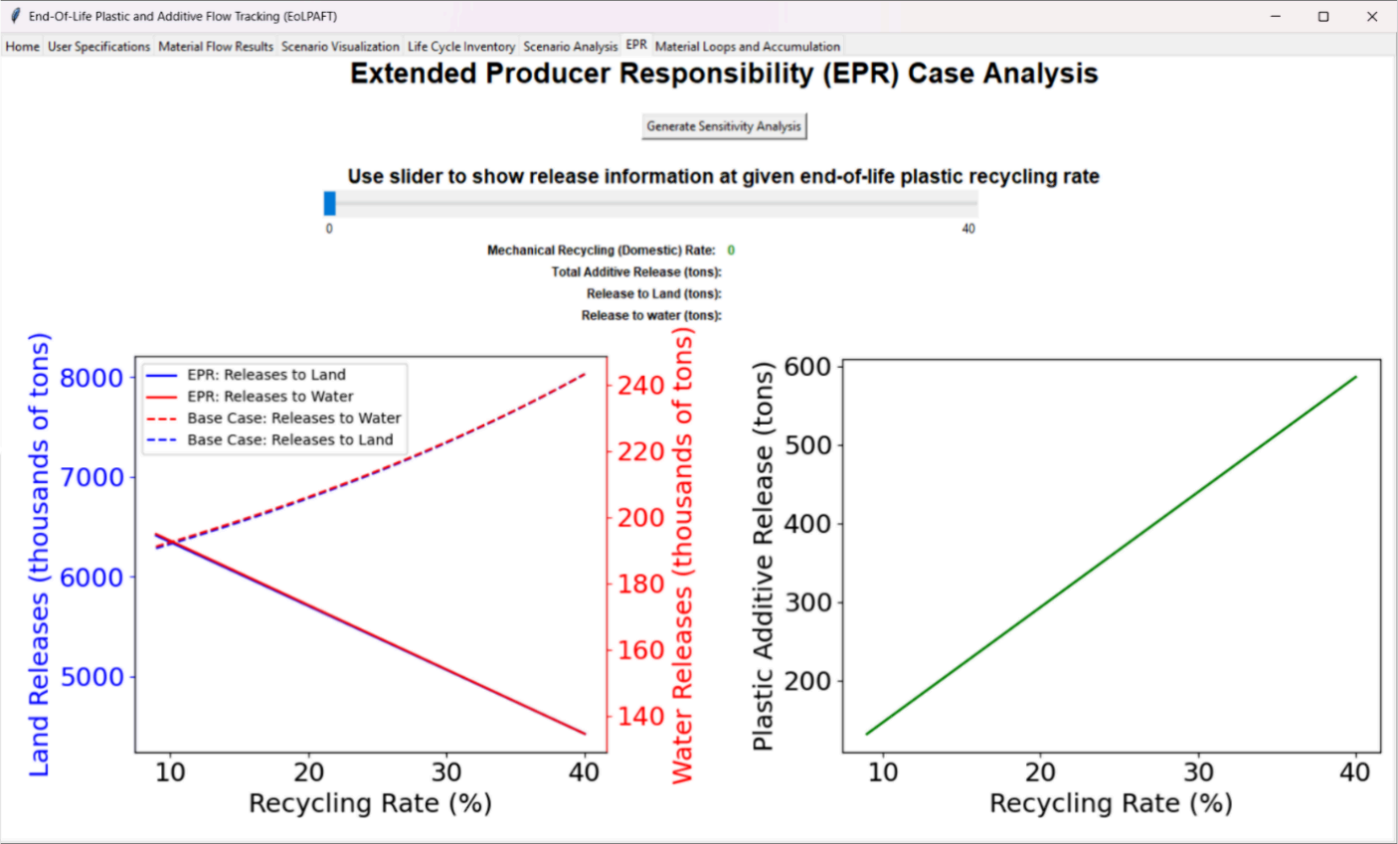
Blue and red trendlines from the leftmost plot display the comparison of the land and water releases (plastics and additives) based on the effectiveness of the EPR program (solid lines) compared with the conventional end-of-life plastic management scenarios (dashed lines). The green solid trendline in the rightmost plot demonstrates the potential chemical additives released into the environment as a function of the plastic recycling rate varied between the 2018 plastic recycling rate and the maximum recycling potential through the Extended Producer Responsibility (EPR) program.

Furthermore, producers are expected to possess the most information regarding the composition and processability of the EoL plastics that they created. However, concerns may be raised regarding chemical additives usually incorporated into several plastic products for enhanced functionality and durability. If subjected to physical processing, chemical additives may migrate from the polymer matrix and be released into the surrounding environment or medium (i.e., food and drinks). The extrusion process can release VOCs, monomers, and other degradation products because of the heat generated from friction.^5^ Figure 5 further shows the potential chemical additive releases by altering the plastic recycling rate exclusively through conventional (mechanical recycling) recycling to the maximum plastic recycling rate expected through EPR program implementation. In the worst-case scenario, the chemical additive release rate increases proportionally with the recycling rate if the EoL plastics are subjected to mechanical recycling as a reprocessing step. The release of chemical additives may be minimized if the alternative plastic recycling methodology (e.g., solvent-based dissolution) is used to maintain the structural integrity of EoL plastics, or the material is reused with minimal processing. However, in most cases, additional treatment steps may be required to remove potentially toxic or unknown contaminants before the EoL plastic is suitable for recycling. Such an endeavor can be cost-intensive and require case-specific economic analysis.^4^

A comprehensive economic analysis at the national level is beyond the scope of this holistic assessment tool at this time. Additionally, market variations throughout the US can make a comprehensive economic assessment challenging. Manufacturers may encounter different investment costs for EPR programs ranging between 0 and 1% of their gross revenue, depending on the specific demands of their markets.^27^ Notably, the federal government assumes no financial burden in adopting EPR programs, with all associated costs shifted onto producers and consumers.^28^ It is crucial to recognize that the success of the EPR framework is dependent on effective collaboration among stakeholders, robust regulatory/incentive frameworks, and continuous monitoring and adaptation to evolving market conditions. Such measures are essential to ensure that the program remains economically viable and sustainable and contributes meaningfully to the circular economy.

Given that the EPR inherently subtracts gross revenue from producers, incentives should be offered to encourage participation. Maine has taken the initiative to pass the EPR program for packaging in July 2021, offering reimbursement to municipalities that choose to participate in improving the recycling infrastructure.^29^ Exemption to the law was offered for producers that made less than $2 million in gross revenue threshold or used less than 1 ton of packaging materials. Oregon shortly passed the same law, which gave rise to the Producer Responsibility Organization (PRO), designed to improve and expand recycling services, including funding waste prevention grants and studies related to recycling systems. Other states that have adopted EPR programs have adopted distinct approaches to determining producer fees and recycling goals.^30^ However, it can be generalized that incentives and exemptions have demonstrated their usefulness in pioneering EPR programs by encouraging participation from producers, driving behavioral change throughout the plastic life cycle, and offering competitive advantages regarding sustainability claims.

### 3.2 The Impacts of Maintaining a Circular Economy

The inherent complexities regarding plastics and additive releases within the EoL plastic management process strongly support the motivation to transition toward a CE model. However, the conventional recycling approach within the US can perpetuate chemical additive migration and contamination across multiple life cycles. The reprocessed EoL plastics may be reused and inadvertently lead to the release of chemical additives derived from the previous life cycles because additives are generally compounded in addition to the original chemical composition.^31,32^ The previous material flow analysis on EoL plastic management in the United States, based on 2018 MSW data, identified that over 35.7 million tons of plastic waste were generated, with only approximately 8.4% successfully recycled.^11^ Figure 6 illustrates the GUI plot output showing a notable trend in chemical additive contamination, with these levels sharply increasing (+0.6%) between the initial life cycles before leveling off toward a more consistent level in the subsequent life cycles (<0.1% increase before reaching a near 0% increase). Therefore, additional treatment steps or alternative EoL plastic processing methods may be necessary to reduce the concentration of potentially toxic chemical additives in recycled plastics. This tracking methodology only aids in estimating the fate of the original fresh plastics and their transition toward becoming recycled plastics over time. The continuous production of new plastic products using recycled feedstocks can inevitably introduce additional materials that may intermingle with the original plastics, increasing the complexity of tracking chemicals that are not generally reported as releases due to threshold requirements.^33^

**Figure 6:**
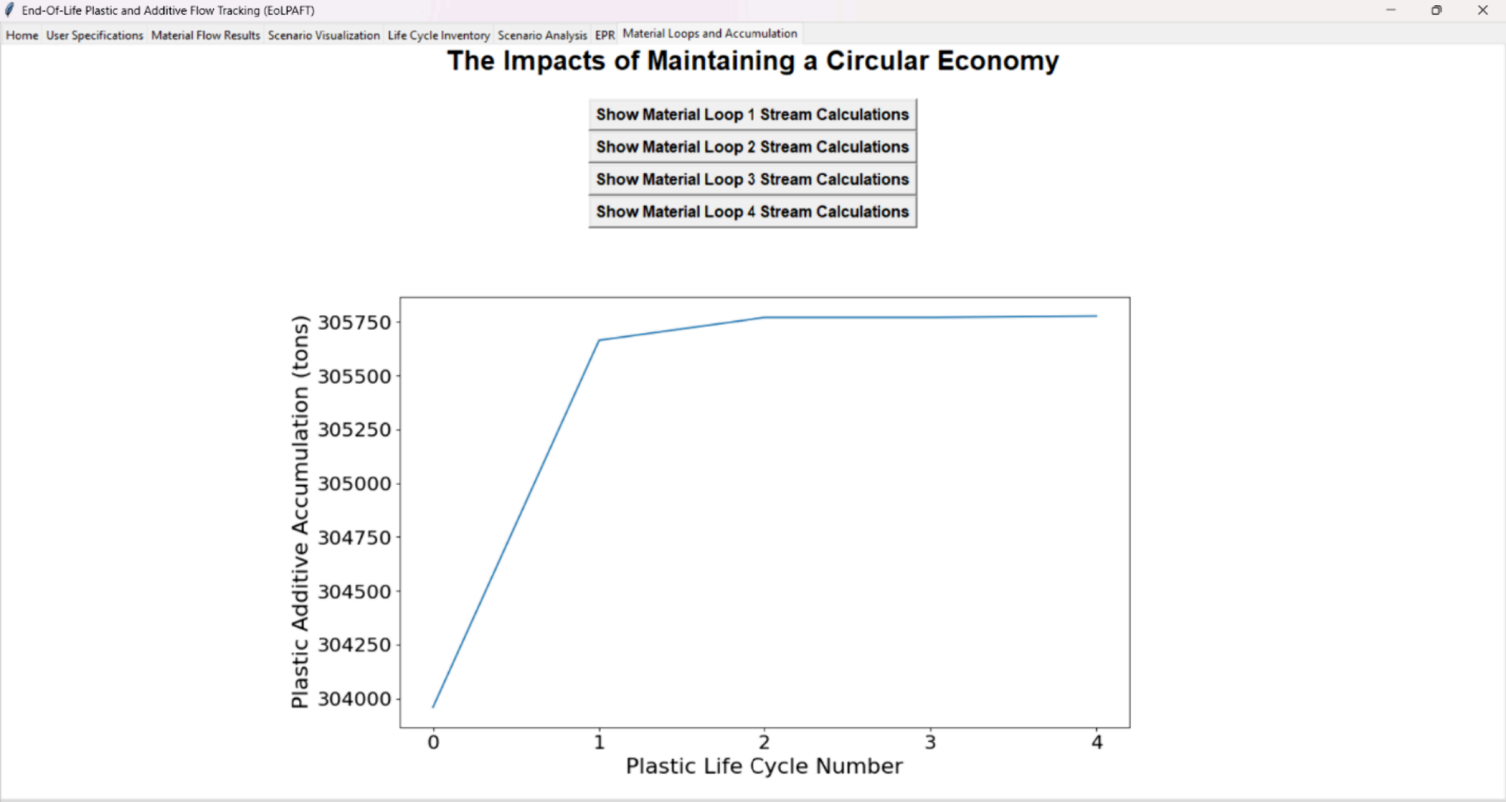
Graphical user-interface display of the potential chemical additive accumulation based on continuous reuse of recycled plastics across multiple life cycle loops

The dispersion of chemical additives into consumer products and the environment originates from the manufacturing stage. Avoiding the use of additives is often impractical because these substances are essential in ensuring desirable properties in the final material for specific applications.^5^ For risk mitigation, plastic manufacturers are expected to have access to confidential business information regarding the formulation of their products. The EoLPAFT can aid manufacturers in understanding the fates of their materials within the plastic supply chain. The potential risks of chemical contamination in plastic products may thus be quantified, creating discussions involving the use of alternative chemicals that may be less toxic and more environmentally benign. Stakeholders and policymakers may also leverage this information to restrict plastic additives associated with health complications, which could simultaneously increase the recyclability and address low recycling rates in conventional EoL plastic management.

The two scenarios presented in sections 3.1 and 3.2 showed that increased recycling as a result of the EPR program could worsen chemical additive releases and accumulations, despite increasing overall plastic circularity. The collected materials through EPR may still be subjected to mechanical recycling, which does not allow for the separation of chemical additives from plastics. Without this separation, continuous recycling across multiple life cycles can increase the concentration of chemical additives in plastic products, thereby increasing the chances of chemical releases to consumer products and the environment.

### 3.3 Uncertainty Analysis

Following the Monte Carlo simulation, the effects of uncertainty in additive release during mechanical recycling for each additive category were found. These results were then normalized and compared to those in the base case, which uses the low mass percent values for each additive category. These normalized results are shown as a heat map in Figure 7.

**Figure 7:**
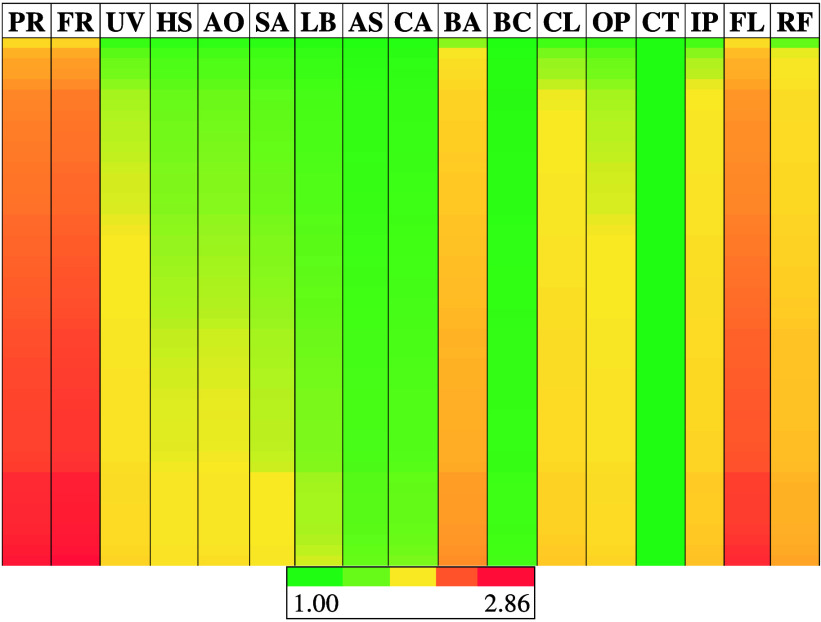
Heat map showing normalized results of Monte Carlo uncertainty analysis. The additive categories are abbreviated as follows: PR (plasticizer); FR (flame retardant); UV (UV stabilizer); HS (heat stabilizer); AO (antioxidant); SA (slip agent); CA (curing agent); BA (blowing agent); BC (biocide); CL (colorant); OP (organic pigment); CT (clarifier/toner); IP (inorganic pigment); FL (filler); and RF (reinforcement).

As expected, the most impactful additives are those that are common to nearly all types of plastics with higher mass percentages and larger ranges in use (plasticizers, flame retardants, and fillers). As a result, these are also the additives whose uncertainty creates the largest range of possible releases. The converse of this is also true: the additive categories found in fewer plastics at lower mass percentages and with a lower use range (clarifiers, toners, and biocides) are the least impactful additives based on uncertainty analysis and overall additive mass. However, it is important to note the overall range that exists: the variation in additive mass composition can nearly triple the estimated additive releases during mechanical recycling, creating a wide range of possible release rates that cannot be narrowed down without further information regarding the plastic composition at large.

### 3.4 Analysis Limitations

The current capability of the EoLPAFT is limited to a material flow analysis, highlighting the overall EoL plastic management efficiency and plastic and chemical additive releases to the environmental compartment with uncertainties. While the results provide a foundational perspective, they do not yet incorporate additional criteria, such as environmental risks associated with persistent, bioaccumulative, or toxic substances (e.g., per- and polyfluoroalkyl substances (PFAS)). The barrier to including these additional criteria depends on the availability of plastic composition data. Much of these data remain proprietary due to intellectual property protections. However, patent literature can serve as a proxy for understanding the reasoning for certain combinations of chemical additive compositions to achieve specific properties for various consumer needs. Incorporating data scraping approaches to extract such information may improve the categorization of EoL plastic fractions, such as identifying those with concerning levels of PFAS, which can encourage the use of safer chemicals during plastic manufacturing and improve the EoL plastic management practices to minimize unintended releases. Despite these limitations, the release estimates of plastics and additives to the environmental compartments reported by the tool can still be inferred to contribute to microplastic and chemical pollution in the environment.^34,35^ Currently, the tool uses published EoL plastic management data from the US EPA, which offers limited flexibility for customization because the data set is primarily used for scenario forecasting.^14^ However, users can still customize the data set and parameters (i.e., relative EoL plastic ratio, plastic recycling rate) to estimate the material management efficiency and releases.

### 3.5 Future Adaptability

Overcoming the aforementioned limitations can allow more complex scenarios to be tested to improve the EoL plastic management methods. Conventionally, physical processing (i.e., mechanical recycling) is used to recover recyclable EoL plastics within the US. However, chemical reprocessing, such as pyrolysis, gasification, and chemolysis, can also be explored as potential alternative processes for achieving a circular economy within the EoL plastic management methods.^36–40^ Chemical reprocessing can handle a broader range of plastic types, including those deemed less recyclable by physical processing.^41^ Pyrolysis, for instance, involves the thermal degradation of heterogeneous plastics without oxygen, producing valuable hydrocarbon products (syngas and oil).^40,42,43^ Gasification converts heterogeneous plastics into syngas at a higher operating temperature than pyrolysis and an oxygen-rich environment, converting EoL plastics into fuels or chemicals.^38,44,45^ Chemolysis depolymerizes homogeneous plastics into smaller molecules through chemical reactions.^41,46^ Before investing in the chemical reprocessing of EoL plastics, considerations must be made for potential environmental releases, energy consumption, and operation costs. Enhancing EoLPAFT to include these chemical processes, beyond the conventional EoL plastic management pathway, would allow users to explore the potential benefits and impacts of these methods, including from a resource perspective. These impacts could be quantified by comparing the viability of adding an alternative plastic reprocessing step to the conventional EoL plastic management scenarios in EoLPAFT.

Incorporating chemical processing as an alternative EoL plastic management option would first require a more detailed generic scenario analysis of the chemical reprocessing of EoL plastics. EoLPAFT currently analyzes high-level material flows through established EoL plastic management processes. There are not enough data available on chemical reprocessing; however, a similar model in EoLPAFT that includes this alternative technology is needed. For example, the heterogeneity of EoL plastics collected can make maintaining consistent feedstock composition challenging, affecting the ability to achieve reliable product quality primarily if the plant feedstock consists of unsorted and mixed EoL plastics. If exposed, the degraded materials can contain chemicals that may harm EoL management workers.^47^ Given that some chemical processing methods (i.e., thermolysis) require high temperatures for material degradation, the energy consumption rate can create an economic barrier to nationwide chemical reprocessing adoption. The economic feasibility relies on factors such as feedstock availability, market demand for recycled products, and regulatory incentives or mandates supporting recycling initiatives. This kind of process data does not exist on a large scale; thus, chemical reprocessing was not included in current versions of EoLPAFT. Still, additional sustainability analyses (i.e., energy, economic, and social needs) to aid in EoL plastic management decision-making throughout various life cycle stages can complement the EoLPAFT and encourage justifiable actions from EoL plastic generators (consumer, commercial, and industrial) to reduce plastic pollution in the environment.

The decision to use Python was made to allow a free and easy distribution of the code with full data transparency. This tool may be customized with new data sets and material flow pathway options as new information and the EoL plastic management landscape change. Additionally, the Python script can be converted into an executable file for users unfamiliar with the coding environment. This accessibility will allow for the inclusion of additional stakeholders, allowing others to engage with the software for better integration of additional data, expert opinions, and further model improvements.

## 4 Conclusions

Addressing the challenges of EoL plastic management requires strategic approaches to ensure maximum material recovery and minimum environmental releases. The Python-based EoLPAFT can help decision-makers analyze the impact of specific plastic EoL management strategies by providing a holistic material flow analysis detailing plastic and additive flow allocation throughout the life cycle and possible releases to the environmental compartments. Information regarding the components of the tool and its installation and usage is available in the SI. The utility of the tool was demonstrated through several case studies involving a simulation of a nationwide EPR program and tracking chemicals of concern through a circular economy.

The EPR program scenario demonstrated an inversely proportional relationship between the recycling rate and environmental releases when EoL plastics are returned to manufacturers for reprocessing. Based on these findings, implementing the EPR programs can improve the US recycling infrastructure and shift plastic management toward a circular economy. However, adopting EPR programs could not eliminate chemical additive release issues, because this release occurs during material recycling, which is a mandatory part of the EoL plastic reclamation process. However, plastic releases were reduced considerably because fewer materials are ultimately processed through conventional EoL plastic management routes. Alternative mitigation strategies for reducing chemical additive exposure risks are required. If recycled materials are reused in the subsequent life cycle, a dispersion of potentially harmful chemical additives may migrate into consumer products, imposing additional health risks. These case studies findings demonstrate how stakeholders, such as manufacturers and policymakers, could use this tool to determine the environmental implications of existing and new EoL plastic management strategies based on the mass allocation of plastics and chemicals of concern. However, implementing these EoL plastic management scenarios requires combined efforts from manufacturers, academics, regulatory agencies, and policymakers to overcome technical, economic, and regulatory barriers and drive innovation toward sustainable EoL plastic management practices.

## Supplementary Material



## Notes

Detailed instructions and walkthrough for software tool use (PDF)
