# Supplementary material for: An End-of-Life Plastic and Additive Flow Tracker Tool for Scenario Forecasting
Source: Ind Eng Chem Res. 2025 Jun 17;64(26):13246–57. doi: 10.1021/acs.iecr.5c00426 (PMC12232299; doi:10.1021/acs.iecr.5c00426)
Supplement: Supplementary file 1 [file ie5c00426_si_001.pdf]

Supplementary Information

An End-of-Life Plastic and Additive Flow Tracker Tool for Scenario Forecasting

John D. Chea[1†], Matthew Conway[2†], Gerardo J. Ruiz-Mercado[1,3*], and Kirti M. Yenkie[2*]

[1]Office of Research & Development, US Environmental Protection Agency

[2]Department of Chemical Engineering, Henry M. Rowan College of Engineering, Rowan University Glassboro, NJ, 08028, USA

[3]Chemical Engineering Graduate Program, University of Atlántico, Puerto Colombia 080007, Colombia

[†]These authors contributed equally

*Corresponding Author: Kirti M. Yenkie, Gerardo J. Ruiz-Mercado

Table of Contents:

Text S1. Requirements... 3

Text S2. Instructions for Use 3

Text S3. The EoLPAFT.py Script.. 3

Text S4. User Specifications and Calculations 3

Text S5. Material Flow Results 4

Text S6. Scenario Visualization 4

Text S7. Life Cycle Inventory... 4

Text S8. Scenario Analysis... 5

Text S9. EPR 5

Text S10. Material Loops and Accumulation.. 5

Text S11. Online Availability and Updates 5

Text S1. Requirements

The required libraries are shown in Table S1 below.

Table S1. Libraries used in EoLPAFT

Required Libraries	
tkinter	matplotlib
numpy	pandas
PIL	xlsxwriter
datetime	plotly
html2image	tktooltip
io	

Text S2. Instructions for Use

This tool can be used to model MSW management systems. The data is entered on the "User Specifications" tab. A reasonable value must be entered into each entry box (reasonable meaning that proportions must sum to 1; calculations will not be carried out if this condition is not met). A "Help" button is available for data descriptions. The calculations can be carried out when all data has been entered, including all the data subsets accessed by the buttons on the left of the screen. The baseline 2018 dataset can be used as a sample, and a "basic" version utilizes this dataset, requiring the user to only input the recycling, incineration, and landfilling rates. Once all data has been submitted, data analysis can begin, and the further tabs in the tool can be used to visualize the data and examine further trends or variations in the data.

Text S3. The EoLPAFT.py Script

The script creates a graphical user interface (GUI) using Tkinter to enable the user to interact with the program. The script creates a GUI window that opens to a set of instructions, with tabs at the top of the screen ("Home", "User Specifications", "Material Flow Results", "Scenario Visualization", "Life Cycle Inventory", "Scenario Analysis", "EPR", and "Material Loops and Accumulation"). Each tab moves the user to a new page and will be described below.

Text S4. User Specifications and Calculations

This screen and section of script uses Tkinter entry boxes and buttons to allow users to input data for the municipal solid waste (MSW) scenario they wish to model. The user can input their own data, or use the built-in 2018 data, as well as modify only certain metrics using the basic version. Buttons appear on the side to allow the user to move between different data categories to complete the input. The data can be "checked" to make sure the proportions are consistent (i.e. add up to 100%). A button submits the data,

and in doing so also checks the proportions. When submitted, the data is stored as a list of dictionaries with keys that are used consistently throughout the entire code, allowing values to be called using their meaning rather than an arbitrary location inside of a list or array.

The function makeCalculations performs the mass balance on the input dataset. These calculations are largely carried out using "for loops" to perform similar calculations on the dictionary datasets that are all formatted identically. Dictionaries group each component in categories such as plastic resins (which includes components PET, HDPE, LDPE, etc.), additives (plasticizers, flame retardants, biocides, etc.), and general municipal solid waste (food; wood; rubber, leather, and textiles; etc.). Calculations are performed stream by stream (e.g. "Manufacturing to Use" and "Sort to Recycling"), and the dictionaries of components in each stream are maintained as a list for access by later calculations.

A "Help" button is also available to aid in a description of the data entries, should the user need further clarification.

Text S5. Material Flow Results

This tab shows the user a Sankey diagram modeling the MSW data they have just input. This Sankey diagram is created using Plotly and is normalized to the overall mass of the system. A button at the top of the screen will take the user to a new window containing a treeview (Tk widget) created from a dataframe which contains all the values from the data calculated above. This treeview presents the actual values of the stream flows that are used to create the Sankey diagram. A button in this new window uses xlsxwriter to export the dataframe to a new Excel book to store the data for the user's later reference.

Text S6. Scenario Visualization

This tab creates a pie graph and bar chart to show the plastic content in the overall MSW and to compare the amount of plastic collected to the amount recycled. A button also allows the user to open a new window showing the specifications input to generate this scenario, with each specification separated into its respective category.

Text S7. Life Cycle Inventory

This tab shows the life cycle inventory of each type of plastic resin and combines all additives into one category. It evaluates the life cycle inventory based on the releases in each phase of its life cycle, and the calculations are again carried out using lists of dictionaries, grouped by phase and then by subcategory inside of that phase (i.e. "Input" or "Output"). The keys of these dictionaries are the types of resins or additives. The data is presented in a treeview widget.

Text S8. Scenario Analysis

This tab carries out the mass balance calculations for all recycling rates up to the rate specified by the user here in the entry tab widget at the top when the "Generate" button is clicked. The additive releases in mechanical recycling and releases to land and water are calculated at each whole percent (i.e. 1, 2, 3, etc.). These releases are stored as a list and graphed. When the user moves the slider, they are selecting a recycling rate whose associated releases values are then retrieved from the list and displayed on the page.

Text S9. EPR

This tab shows the "Extended Producer Responsibility" scenario (EPR). In it, the mass balance calculations are carried out again at different recycling rates, but the 40% of plastics that are packaging plastics are removed to model the scenario in which producers reclaim that waste. Much like the scenario analysis above, the calculations are carried out at different rates of recycling, the values are stored in a list, graphed, and, when a recycling value is selected by the slider, the associated releases value is displayed on the screen.

Text S10. Material Loops and Accumulation

This scenario examines the impact of recycling on additive accumulation. The calculations are carried out nearly the same between each loop, except for an additional component of recycled plastic resins and additives. These are added in and quantified separately (i.e. recycled plastics treated separately from virgin plastics). The recycled plastic is carried over from the previous material loop (so material loop 1 includes recycled plastic from loop 0, loop 2 includes recycled plastic from loops 0 and 1, etc.). The accumulation shown is the total mass of additives in the plastic, which is shown to increase from loop to loop because the recycled plastic brings additives with it.

Similar stream values to those shown in the "Material Flow Results" tab are available here for each material loop in a treeview, and can be exported to an Excel book in the same way.

Text S11. Online Availability and Updates

Link: https://yenkiekm.com/computational-modules/

Download Link: https://github.com/jdchea95/EoLPAFT
